# Internalizing Knowledge and Changing Attitudes to Female Genital Cutting/Mutilation

**DOI:** 10.1155/2013/467028

**Published:** 2013-06-12

**Authors:** Inger-Lise Lien, Jon-Håkon Schultz

**Affiliations:** Norwegian Centre for Violence and Traumatic Stress Studies, Kirkeveien 166, Bygning 48, 0450 Oslo, Norway

## Abstract

The process of paradigmatic attitudinal change has been analyzed by the use of multimethods and multileveled internalization theories. Forty-six informants (a network of activists and a group of Gambian women) have described their change of attitude to female genital cutting. This study shows that internalizing a packet of information as adults, that contradicts an old schema of knowledge internalized as children, can be experienced as epistemologically very painful. Activists in Norway who have changed their attitude to FGC have got information from different educational institutions, from seminars and conferences, from work as interpreters in hospitals, and from discussions among families and friends. Information can be received, listened to and subsequently discarded. In order to design FGC-abandonment campaigns, the importance of the internalization process in order for the individual to make an attitudinal change must be understood.

## 1. Changing Attitudes to FGC

Female genital cutting (FGC), also called female genital mutilation (FGM) and circumcision, takes many forms, from milder to more serious procedures. The World Health Organisation (WHO) has divided these into four types [1]. (1) *Clitoridectomy* involves partial or total removal of the clitoris and/or the prepuce. (2) *Excision* involves partial or total removal of the labia minora and/or the labia majora. (3) *Infibulation* involves narrowing the vaginal opening through the creation of a covering seal with or without removal of the clitoris. (4) *Others* involve all other harmful procedures to the female genitalia for nonmedical purposes. The procedure are mostly done on girls from infancy to 15 years of age. In many countries girls are being cut at earlier ages than before [2]. Internationally FGC is recognized as a violation of human rights as it can have serious short- and long-term effects on the heatlh and well-being of girls and women.

In spite of many abandonment campaigns through years, it has proved difficult to abolish the practice [3]. There has been “greater success in raising awareness about the issue,” Shell-Duncan and Hernlund [4] say, “than in changing behavior.” Medicalizing the procedure by having it done in a hospital by health professionals was intended to alleviate some of the pain related to FGC, but it may have encouraged the practice instead of abolishing it [5]. A systematic review of the effectiveness of FGC prevention interventions [6] evaluated interventions [7–11] that ranged in length from two hours to two years. The review concluded that the evidence within such studies was too weak in order to base a solid conclusion to what worked and what did not. Information spread in campaigns consisted of everything from role playing, health talks, theatre groups, community meetings, and videos to educational sessions in human rights and hygiene. After a two-hour course at a university in Egypt [10], for example, the students had learned about FGC and changed their attitudes to it, whereas the attitudes of health personnel in Mali [12] did not appear to have changed after two months of education. Why? Such differences have been difficult to explain. Although there has been a change in prevalence in some countries, due to these campaigns, in too many countries, there has been very little change in the frequency of FGC [1]. 

Migration has made many European governments concerned as it is suggested that more than half a million women and girls have undergone the procedure or are at risk within the European Union [13]. Laws have been passed; abandonment campaigns and action plans are implemented [14, 15]. According to Johnsdotter [16], the enabling environment in countries where FGC is condemned is central to the rejection of the tradition. This was true of Somali immigrants in Sweden. In a study of 33 Ethiopian and Eritrean men and women in Sweden, Johnsdotter et al. [17] found that they rejected all forms of FGC. Gele et al. [18] studied 38 Somali men and women in Norway and the majority of their informants said they would not cut their daughters. Living in an environment where the tradition is illegal and associated with low social status can change people's attitudes, the authors argue, a finding that Talle [19] confirmed in her work among Somalis in Norway. The above mentioned studies, however, say nothing about the way information is received and processed by individuals and groups. This study interviewed activists to obtain an idea of their reactions to information provided at seminars and conferences. The idea was to find out what triggered a strong commitment to abandon the practice so that it had an effect on both attitudes and behavior. Some of the informants of this study had been living in Norway for years before getting the information that caused them to change attitudes and behavior. So what triggered for change? And how did the informants internalize the information? We need to understand both the social and individual processes that work when people are exposed to reeducation campaigns. Without this information it will be difficult to design programs with the ability to change values that people feel are meaningful [20] as well as their behavior.

This paper seeks to describe and analyze the way in which persons who were socialized in a cultural context where FGC is highly valued receive and process information that contradicts and devalues the meaningful norms and traditions they internalized as children. As has been described by Schultz and Lien [20], norms and values related to the practice are deeply internalized by children through metaphors in a meaning making process that deals with morals, knowledge, and esthetics. As Schweder [21] has argued, unmodified genitals are seen as ugly, unrefined, uncivilized, and not even human. To change from this perspective to seeing unmodified genitals as natural and human, one has to take a completely different view that we in this paper will describe as making a paradigmatic change of attitude. Kuhn [22] sees a paradigmatic change as abrupt, based on an epistemological crisis within a conventional mind set. An example of a paradigmatic shift in science is from a geocentric model, where the earth is the center of the universe to the Copernican worldview where the sun is the center. A paradigmatic shift like this, Kuhn argues, is so fundamental, that knowledge from the first paradigm cannot be transferred to the next. He calls this *the incommensurable thesis*. When a new paradigm is formed, it will have new followers, and an intellectual “battle” will take place within the person, but also between the followers of the new paradigm and the holdouts of the old. Reasons for practicing FGC, what we can call “the old paradigm,” vary across different social contexts, but often include magic (touching the clitoris can lead to death of the children and spouse), esthetics (after FGC, the female body is clean, hygienic, and beautiful), morality (the woman remains virgin, chaste, and virtuous), and social esteem (the woman retains her honor and will more easily find a husband) [23–25]. In order to understand a paradigmatic shift of attitude from an “old” to a “new” packet of knowledge and meaning, we need to understand the internalization processes that take place within individuals and the processes taking place between individuals and groups.

## 2. Internalization Processes and Change

In his analysis of innovation, Rogers [26] identified five main steps in processes of attitudinal change: (1) knowledge, (2) persuasion, (3) decision, (4) implementation, and (5) confirmation. He distinguishes between different types of adopters: early adopters, late adopters, and those who are very late; he altogether operates with five types of adopters. According to Rogers, it is easier to adopt an innovation that is consistent with existing values than one that is not compatible and requires, according to him, a new value system in order to be adopted. The model of diffusion of innovation deals with actors, systems, structures, networks, time, and space and has relevance both for developing programs and analyzing attitudinal change with respect to FGC.

Mackie [27, 28] is concerned with the change of conventions and has tried to identify the tipping point necessary for a conventional shift of behavior and attitudes to FGC on an aggregate level. The theory has injected much needed energy into the abandonment campaign. The use of public declarations, recommended by the theory, seems to have worked effectively in changing behavior in several African communities [29]. However, the depth of a conviction and the thickness and complexity of a convention are not adequately dealt with. As it is a macrolevel theory, it does not pretend to deal with how people internalize information on FGC that can contradict entrenched and cherished norms and values.

In analyzing social change in respect of FGC, Shell-Duncan and Hernlund [4, 30] focused on the decision-making process itself, not at the aggregate or individual level, but the mediate group level. The decision to cut a girl often lies within an extended family or group, they argue, thereby reducing the insights that an individually based theory of change can provide. In their view, attitudinal and behavioral change does not necessarily proceed in a linear fashion, but is fluid and more shifting. There are five decision making dimensions of readiness to change: (1) supporters of the continuation of FGC; (2) those who contemplate abandoning FGC; (3) reluctant practitioners of FGC; (4) willing abandoners; and (5) those who have reluctantly abandoned FGC. Thinking, saying, and doing represent different levels of decision making and are integrated in the theory. So their main focus is the decision-making process itself, not the process of internalization or diffusion.

Interests into internalization theories have increased, something that several researchers have welcomed [31–33]. Spiro [34] was among the first anthropologists to examine internalization theoretically; he views individuals as active participants in the process. For a cultural proposition to become a culturally constituted belief, it must be acquired at one of four levels of conviction [34]. The first level is when a cultural novice becomes acquainted with the proposition, without assenting to it. At the second level, the novice acknowledges the proposition, but does not internalize it. It is received as a cliché. So according to this theory, a person can be informed about the health risks of FGC, consider this information carefully, but ultimately reject it because s/he finds it implausible or inconceivable. It may come across as Western propaganda, and while it awakens a curiosity it is also met with disbelief. It is only by the third level when the cultural proposition has been accepted by the person, that it is internalized. And at the fourth level, it becomes a genuine belief held to be true, proper, and right. We may argue that it is only when a package of knowledge reaches the last two levels and becomes motivationally saturated that a paradigmatic change of attitude will be made.

Lawrence and Valsiner [35] propose a model of internalization, in which they draw on anthropological and psychological theories like the previous one. There are three layers of internalization, they argue; information at the last level becomes integrated into existing knowledge and emotional structures. The mechanism by which information is internalized is self-talk, and the message is taken in and processed selectively and met with barriers and buffers. Information can be discarded through an inner dialogue. When information has reached the final level, it is transformed and communicated into the social world by externalization: “…human beings are social not because they conform mechanically to external social expectations, but because they transform these expectations into their own personal-cultural, intrapsychological inventions that are functional for their further encountering of the world” [35]. Change, then, comes from processing and dealing with the novelty created in encounters between people, and between people and the social system. The human mind is both social and personal inasmuch as it is active in internalizing information with the aid of external social institutions and persons. By means of these internalizing and externalizing processes, meaning, as well as innovation, change, and predictability in social contexts, are created and spread. 

Internalization models are useful for analyzing and understanding the appropriation of cultural meaning through socialization of children. But it is equally important in order to understand social change when new information is internalized by adults. FGC abandonment campaigns offer excellent cases for study because change takes place both at the personal and collective level as women move from one context to another, where information is provided that may contradict knowledge acquired earlier. As we have seen from these theories, information may be met with resistance and not internalized so that it will motivate action, but received as a cliché and stopped at the first or second level. In a study of FGC among Somali women in Norway, Johansen [36] found that some of the 45 women she interviewed had begun to question the tradition, and some were preferring milder forms (sunna), which they did not classify as “genital cutting.” This allowed them to retain the customary system of justification. The changes were modifications within the existing paradigm, indicating that some information within the new context have been received and internalized without this resulting in a *total* paradigmatic shift of attitude.

But what happens when women get information at a deeper level that makes them adopt a new model of thought? 

## 3. Methods

Our study is based on a multimethod approach in order to provide us with knowledge from observations in seminars, individual interviews, and focus group interviews. The intention has been to get both individual and contextual data. Anthropological field work was the method we started out with. Over a three-year period, we have been visiting Gambian and Somali families in Norway and taking part in social events in order to understand the context and meaning of FGC in their everyday life. We took part in conferences and seminars in Norway that were arranged on FGC by the Norwegian government and seminars that were arranged by nongovernmental organizations. The reasons for attending seminars were to observe women's reactions to information given and to get complementary knowledge on what seemed to work and what did not. We also followed a group of Gambian women that used to meet each other once a month. In 2008, a Gambian couple, was arrested for having sent three daughters to Gambia in order to have them cut which upset this particular group. We arranged two focus group meetings with these women, where more women than planned turned up (twenty persons present). We also interviewed the activists individually about their shift of attitude to FGC. In a context where FGC is criminalized, it can be difficult for a person who supports FGC to talk honestly and openly about the attitude. Working actively against FGC is an indication of a high level of commitment against the procedure, indicating that the person has internalized new information and made a paradigmatic change of attitude that has had behavioral consequences. We applied the theory of internalization methodically by identifying indicators of internalization. By listening to individuals' and groups' own description of how they received information, we could identify to what degree they reflected on information, processed it through talk, believed in it, or became committed. Emotional reactions, holding information credible, and becoming motivated are the criteria by which we judged their level of internalization.

The 26 anti-FGC activists (aged from 20 to 58) belonged to a network of women who used to meet at seminars and conferences. They were from Eritrea, Ethiopia, Gambia, Ghana, Kenya, and Somalia. The selection criteria were involvement in anti-FGC campaigns; participation as speakers at seminars and conferences arranged by the government; published articles and/or books against FGC; interviews with newspapers; membership of working groups; acting as adviser to the Norwegian government. The activists were interviewed twice. The interviews were focused on the process of changing attitudes to FGC, key events triggering attitudinal change, and response to new information. The interviews followed an open explorative design, where we started with questions about their attitudes before (the old paradigm) and now (the new paradigm). We asked them to describe, step by step, their process of change, and how they felt about it. Did it happen suddenly? What triggered the change? And what happened after the change? The researchers performed the interviews in Norwegian or English, sometimes alone, but mostly together. In some cases, we used a microphone, but mostly we performed the interviews without it. As we were making parallel notes, we could compare them and discuss any differences, a procedure which served to validate the data. If anything was unclear, we would could call back the person in question for another interview. Some respondents were therefore interviewed three times. 

The cases presented in this paper can be called “apt illustrations” [37], inasmuch as they capture elements and insights which might be overlooked in a survey. 

## 4. Descriptions from Two Seminars on FGC with Gambians

We will start our presentation of the data by presenting material from two seminars that a group of Gambian women arranged. The government had not targeted the Gambian community FGC-related information. Some members of this group told us that they decided to arrange the first meeting on FGC after the arrest of the couple, to “calm down the government.” 

An invited guest from an English organization, an African Muslim, talked to a hundred Gambian women and men in Oslo about FGC for an hour, described the four different types, and explained health consequences both short term and long term. There were several reactions to the lecture that we will present here: “she called it FGM and told us horrible things.” Another: “it is incredible that a grown up women can stand there and lie to us all.” A key informant that later became an activist said the following about the first seminar:

*“We thought it was a joke and laughed. We were all very happy that we had healed after circumcision, and did not suffer like the Somalis. We were in a state of denial. It was later, when I started to read the government's pamphlets that I understood that there were many things I did not know.”*

Later, during the two focus group interviews, the researchers were told that most of the women did not believe in the information that was given during the first seminar. This can indicate that information was received at levels one and two, where it was put to a halt and met with resistance as it was not believed to be credible. It was heard and memorized, but it was not processed and internalized further down to levels three and four so that it made an impact and became motivationally salient. As researchers present at the seminar, we could see how the women acted: looking around, fiddling with their hand bags, whispering to the next person and trying not to pay much attention to the information given. 

Three months after the first seminar, a second seminar was held, arranged by Gambians with an invited activist from the Gambia giving a lecture about consequences of FGC. There were more than a hundred Gambians present in a lecture hall. A Gambian woman with a university degree, having lived in Europe for more than 20 years, told us that she would have cut her daughter if she had not attended this particular meeting. She said the following:

*“The Gambian woman, a doctor, asked whether she could show us some pictures of girls' genitalia. We protested, and this made her angry, but in the end, she managed to convince us to watch the slides of small Gambian girls suffering from conditions due to FGC. They had scars and cysts. Others had fistulas. We could see that the children were in pain. We were shocked. The Gambian doctor sang the secret songs from the circumcision ritual. That shocked us even more. The tension in the room was electric. There were tears in the eyes of every Gambian woman present. I got gooseflesh, all the hair on my body stood out. We realized that she herself had been there, that she herself had been circumcised. It was an emotionally strong discovery and experience. I understood, there and then, that this tradition is definitely wrong.” *

As we can see, verbal and visual information on the adverse consequences to health, seeing pictures of small children in pain, and hearing songs which reminded the women of their own cutting had a very deep impact. It took them further down the steps towards internalizing information that they may have begun three months earlier. The events during the second seminar sparked individual reactions. For some persons, the information went to the last levels and became motivational. For others who were present, information was not deemed as credible and reached the first two internalization levels. As one person said “a log can stay in the water as long as it will, but it will never become a crocodile.”

To a greater extent, the women could identify with the invited lecturer in the second seminar. She had authority, she was a doctor, and she was from the Gambia and circumcised herself. Verbal information was supported by pictures, slides, a video film, and a song. Present in the lecture hall, we as researchers could observe that several of the women had tears in their eyes when the doctor was singing the secret song from the ritual. Through these varieties of means, the whole person was targeted, both intellectually and emotionally. This enabled information to sink in, so that, in the end, there was a group of women leaving the seminar who had changed their attitude to the issue. 

Twenty of the Gambian women who had taken part in both seminars were interviewed in a focus group about their reaction to what they had heard and seen during the two seminars and otherwise. Among them, 18 had been very much in favor of FGC until the last seminar, which changed their opinion. Two had been against FGC from the day they were circumcised. The reasons given for change were the slides of girls with severe FGC-induced health problems and the song, which moved them and brought memories back of their own cutting. Information on health risks and human rights was also mentioned. An Imam had also said that FGC is not a religious injunction, which was also mentioned to be important.

The women were to describe their emotional response to the information provided at the last seminar. Here are some of their remarks. “I cried inside,” “I was sad,” “I had a feeling of the same powerlessness that I felt when I was circumcised,” “I felt paralyzed,” “all energy disappeared,” “I felt guilty,” “I got angry,” “I lost pride,” “I thought I shall not circumcise my daughter if I get one,” “I was sweating,” “my heart was beating,” “I got gooseflesh,” and “I was numb.” As one woman said “when I got information at the second seminar it was as if all the energy in my body disappeared. As if I was fainting. It was terrible.” 

## 5. The Activists

It was after these seminars and focus group with the Gambian women that we decided to interview 26 activists about their personal change of attitude to FGC. We found that they expressed more or less the same emotional response to some key events that got them to change their mind about FGC. They had changed their view especially when hearing about health consequences and seeing slides. When describing the inner feelings, they used words like shocked, angry, sad, depressed, shameful, they wept, were scared, provoked, unhappy, paralyzed, and pained. The frequent use of these words to describe their emotions indicates how psychologically painful it can be for many women to allow information about consequences of FGC to make an impact at a deep psychological level so that it changes their attitudes. Here is a Somali activist's description of her process of change:

*“I turned against FGM when I was attending anatomy classes in physiology when training to become a nurse in Somalia. Our teacher was from Beirut and one day she explained to us about genital mutilation. She used slides and explained about the natural genital organs. We thought she was crazy. I had thought all women all over the whole world were circumcised—and now she was saying they were not. In the evening the girls sat together and started to cry. The next day the teacher continued. She told us about the complications, psychological and physical effects. Now the girls understood why they were in pain. We talked about it the whole day. I understood that this is something we must work against. Other girls said that we could not work against it because it would be too difficult in Somalia.*

* When you are circumcised you are proud, and I preserved my pride until I was 18. I lost it when I got the new knowledge. When I spoke with my friends about it, we were not proud anymore. I started to mourn about my body. I comforted myself with the fact that it had happened and there was nothing I could do about it. But I promised myself that it shall not happen to my daughter. I started to work with the issue with UNICEF and the Red Cross. That was before I left Somalia and came to Norway.” *

Here we see how information was dealt with in a collective class situation; it was through talking to oneself and others that information was processed, and as it moved down the levels, it involved emotional reactions, crying, and mourning. Talking with others could have both an internalization effect, leading to reflection and analysis, and a therapeutic effect. The pain associated with new information could be dealt with through these collective processes, but the pain factor can also explain why information can get to a halt when it comes to internalization, so that it stops and/or is rejected at levels one and two. 

It can take time to move between levels. A woman described how surprised she was arriving at an asylum center in Norway, meeting other Muslim women that were not cut. She reacted with disbelief and surprise, memorized the information as a curiosity until some years later she began to study in a nursing college. Before this, she had studied in a high school, where FGC had not been a topic that was discussed. At the nursing college, however, she got information “…that made me fall into a deep depression. I used to be proud of my circumcision. But the information made me suffer, and I suddenly came to see myself as a victim of a terrible tradition.” So here again we see that information can lead to psychological pain, and there can be a time gap within the internalization process, particularly between the levels two and three. 

### 5.1. Key Events

The activists mentioned four types of incident that made an impact on their attitude. (1) Formal education: six persons mentioned health training, especially in anatomy classes. (2) Concrete experience of working at a hospital: four women had worked as interpreters at hospitals in Norway and saw infibulated women giving birth. Working alongside and talking with health personnel, who said that they had never seen an infibulated woman, was an eye-opener and changed their attitude to the practice. (3) Seminars and conferences, nine persons mentioned that attendance at seminars, conferences, and membership of working groups as the key events made them change their mind. These meetings were arranged by the government and different NGOs. (4) Discussions with family and friends: one woman, whose husband was a medical doctor, said it was what he had told her that caused her to change her attitude. Other activists had met people from their own background who were opposed to the practice and who convinced them that FGC is wrong. Many of them had gathered what Norwegian law said about FGC and had pondered the risk of imprisonment. The combination of information from several quarters may also have made a complementary, aggregated contribution and brought about attitudinal change.

## 6. Discussion

### 6.1. Internalization of New Knowledge

Cultural ideas in favor of FGC can be described as a specific cultural model [38], as a cognitive schema that has motivational force [31, 38], as a package of knowledge [39] that has deep cultural meaning [32], or as a paradigm [33]. Mackie [27] describes the shift of attitude to FGC in terms of breaking out of a “belief trap.” 

Adopting a new system of thought on FGC involves seeing the tradition as bad instead of good, as negative for female sexual identity instead of positive. As Shell-Duncan and Hernlund [4] noted from Gambia, an uncut woman is labeled as a *solema*, a highly derogatory term meaning not only uncircumcised but also “rude, ignorant, immature, uncivilized, and unclean.” Information that accompanies abandonment campaigns is often based on scientifically produced knowledge which concludes that FGC does not have the beneficial health effects that the holders of the tradition claim, rather the opposite. Indeed, it can be difficult to transfer information that is valid under one system of thought to another without difficulty. It may require the structuration of a new schema within the mind, so to speak. One activist said “I used to be a proud woman, but when I learned about FGC, I lost my pride and came to see myself as a victim of a harmful tradition. I fell into a deep depression, and cried. Then I started to work against the tradition, and was proud again.”

In an analysis of “moral revolutions,” as in the changing of attitudes to foot binding in China, Appiah [40] argues that honoring and shaming are the motors behind this change. As a consequence of internationalization, China has included outsiders as relevant partners for discussion and thereby become dependent on them as a public that can recognize their traditions or not. The condemnation of foot binding that came from outsiders has been listened to, creating feelings of shame, and shifted their conceptions about what is honorable. As a Chinese informant said, “there is nothing which makes us objects of ridicule as much as foot binding. I, your humble servant, feel deeply ashamed at heart” [40]. The Chinese expanded their public to include oustiders who opposed the tradition. They internalized information that came from this group in spite of the fact that the information caused the painful feeling of shame. 

As our informants have shown, simply living in an environment where information floats randomly around does not necessarily cause attitudes to change completely. The sender of the information needs to be recognized and given authority for information to be listened to and fully internalized. A process of recognition of outsiders seems to have taken place in China, leading to a change of attitude. There are, however, several ways of discarding information: by not recognizing the authority of the group of senders; by denying the credibility of the knowledge itself; avoiding settings in which information is given; because information campaigns do not target the target group to which one belongs. 

Information, then, is not always absorbed; it can be ignored, and it can be met with barriers and resistance. When information is believed and taken serious, it may reach levels three and four (within Spiro's theory) or level three (in Lawrence and Valsiner's theory). When information is resisted and disbelieved, it will be received as a cliché, and it will only reach the first two levels. In spite of abandonment campaigns coming from the government, many of the Gambian women, having lived in Norway for years, did not seem to have received information or had received information at a superficial level, or not taken it in at all. Several of the women in the focus group said they would have cut their daughters if they had not attended the seminars at which African women made the case against FGC. Deeply internalized information can get women to promote and spread the new ideas. They transform them and get involved in a process of externalization [35], diffusion, and confirmation [26]. The idea that there are levels within a process of internalization helps us understand how information disseminated by governments and NGOs can have little lasting effect and do little to persuade people to abandon FGC, if the sender has no authority with the audience, or if the group of women being the target for the information is not at all interested or motivated to take it in past levels one and two. 

When conflicting information is internalized, Strauss and Quinn [33] suggest that it can be solved either mentally through rejection, by establishing a partly integrated separate schema in the mind, by making an unconscious compromise, or through both mental and social compartmentalization. Motivation associated with the schemas can also vary, as implied by Spiro's [34] internalization dimension. 

A Gambian woman explained in her own way how speech can be anchored at different internalized levels and stated: “some women will talk to you about circumcision from their throat and not from their deepest heart.”

### 6.2. Reaching the Last Level

Bateson [41] used the phrase “hitting bottom” to represent the stage that must be reached before it is possible to stop drinking. “Hitting bottom,” says Bateson, represents a turning point at a deep psychological level. At this bottom level, the person makes a “paradigmatic” shift of attitude to alcohol. Even though drinking is not a perfect analogy for FGC, the formulation of “hitting bottom” is a useful metaphor for describing the deep epistemological change that occurs when deeply held attitudes change paradigmatically at the bottom level described in the theories of internalization. As Bateson has described for the alcoholics, it involves both a loss of pride and a feeling of shame that are painful. We see a parallel with FGC where women, on “hitting bottom,” seem to lose their pride, feel ashamed, and start mourning. This process of change, from honor to shame, has been described by Appiah [40] as part of a “moral revolution.” It is not only a loss of personal pride, but of pride in a cultural identity. As one of the women said, “I had to recognize the fact that my forefathers, my people, had been so stupid, so incredibly stupid that they have put their daughters at risk for hundreds of years, unnecessarily.”

The new information often comes from another system of thought, from people with another cultural background. The outsider may not be recognized as an authority, which can prompt people at an early stage to refuse to believe what they are told. The information can be received only at the first or second level. 

In the case of the Gambian women in our case material, the information that convinced them came from a Muslim woman from their own cultural background, who was cut herself and who sang the cutting ritual song. The first seminar only made the Gambian women feel skeptical. The messenger was a foreigner living in England, not a Gambian, but a Muslim, and mostly talked about the Somali customs. Her information was deemed irrelevant, and not credible, but may have paved the way for a change of attitude three months later. At the second seminar, however, they reacted with shock, resistance, mockery, and denial, but then, several of them accepted the information. 

Writing on the epidemiology of representations, Sperber [42] argues that “authority” creates susceptibility for representations not fully understood. One woman described how she felt about the information: “it came from her, a Gambian and a doctor, and was not Western propaganda, it was real.” By singing, the doctor had revealed that she was a cut woman who knew the secret song of the cutting ritual. This created empathy, credibility, and trustworthiness. Informants later said that the singing reawakened their memories of the ritual chamber. “Sitting there, I could even remember the smell.” Another said “I could feel the same powerlessness that I felt as a child when my arms and legs were held during circumcision.” Verbal information in this case was accompanied by and strengthened by nonverbal, visual, and metaphorical information that managed to pierce the barriers and levels, sinking down and moving the emotions.

According to Lawrence and Valsiner's [35] theory, it is at the inmost layer (layer III) that a material becomes integrated into existing knowledge and emotional structure and made part of a critical dialogue with oneself. Through this dialogue, information is accepted or rejected. One might expect people to accept only such information as is compatible with already existing information. But this is not what happens when people open up to new information about FGC. 

The newly internalized knowledge represents an interpretive frame that can be used to illuminate and reflect over old knowledge. It can lead to an interpretation of the “old” paradigm so that it becomes shameful instead of honorable. The “new” paradigm represents an alternative, allowing new questions to be posed. What passes in one context for good parenting can, when seen from the point of view of another schema of interpretation, be subject to interrogation in a completely novel way. As one of our informants asked rhetorically in an accusing voice, “why did you do it, mom?” The relationship itself, between mother, daughter, and grandmother and the morality of the tradition, was reassessed. What was held as moral became immoral, what was justified became unjustified. The old, deeply internalized and embodied knowledge was questioned and defeated. It caused epistemological pain and would require the reorganization and reconnection of the schema within the structure of the mind. Strauss and Quinn [33] see the compartmentalization of information within the mind as a strategy to solve a conflict between values or schemas, a split mind and/or ambivalence, or a restructuring, creating a hierarchy of values and schemas.

This process of connection, reconnection, disconnection, and transformation of information, we think, is a way of dealing with the pain. The relationship between internalized knowledge and the motivational force it carries can also be changed. Motivation can change in an instant, something we saw several examples of, or it may take time, as we also have seen, undergoing a process of confirmation by talking with oneself and others, that would require that the person takes part in several seminars and conferences. The social process itself will pressure the person to find a solution to the conflict of values, which can be prompted and expressed in rejection, transformation, the making of new meaning, and restructuring of old information. Some of our informants blame imperialism, which happened a long time ago. As one Somali woman said, “it was originally a foreign tradition that entered our culture, we thought it was good, but it was not, so let us take our culture back and abandon FGC.” A way of creating new meaning related to parental caring was also expressed by several women: “they were ignorant and did their best. We cannot blame them. They tried to be good parents.” 

Newly internalized information is thereby connected to preexisting core values such as caring for children. According to Mackie and LeJeune [28], the basic values of parents everywhere involve caring for their children and protecting them from any harm. When parents receive new information, for example, about the harmful effect of FGC, they do not necessarily change their most basic values, but “realize their basic values more coherently and more fully” [43]. Through a process of disconnection, transformation, and reconnection, knowledge is weaved into the internal structure, creating new meaning and a proud sense of self. Information is transferred into the world by externalization, which can involve activism. 

## 7. What Happens after “Hitting Bottom”?

All the 26 activists who were interviewed individually had started to work against the tradition by lecturing at conferences, seminars, and in small group sessions. Those activists with a high media profile have earned the admiration of their Norwegian audience, but seem to be paying a price within their community. They report being laughed at, harassed, having stones thrown at them, being excluded, accused of spying for the government, and destroying their culture. This suggests that the new knowledge that has been internalized at the deepest level has been combined with a strong motivation to express that knowledge in spite of the condemnation of parts of the community. Two of the Gambian women remain supporters of cutting, but five became activists establishing an NGO to combat FGC. The work involves other Gambian women in the group who make tea, serve cakes, or register participants at meetings and conferences. 

Their activism has given them new meaning, more respect, and higher status. A few had received awards; they belonged to new networks with new contacts and felt a greater sense of integration in society. Activism can also give new meaning as a substitute for the loss of meaning. 

The cost of a paradigmatic change of attitude, or change of schema, may, however, seem too high for some, and they may choose to keep the new attitude a secret, pay lip service, and compartmentalize. The problem of backsliding, or having to act against one's own principles when one is alone in one's convictions, or exposed to family and social pressure, indicates that interventions should target groups within the community, in a Western setting. The importance of talk in order for information to be internalized and confirmed has been demonstrated. The sharing of knowledge with others within communities and groups will work therapeutically and mitigate the epistemological pain; it can also make the new knowledge more sustainable. Lessons from Africa using official declarations can also be used within seminars and conferences in the West creating group solidarity, commitment, new meaning, and work as a symbolic marker for both collective and individual changes.

## 8. Summary and Conclusions

In this article we have described and analyzed personal accounts of changes in attitude to FGC using a multileveled internalization theory. We have shown that new knowledge that challenges old knowledge that has been taken for granted can be painful. Internalizing information at a superficial level can also cause people to resist and/or discard information or relate to it in a superficial manner. Information internalized at “bottom” level, on the other hand, can generate new attitudes in spite of the fact that it is painful. 

Most of our informants changed their viewpoints after attending seminars, conferences, or training courses. Social and attitudinal change can happen slowly or in an instant. Internal psychological processes and social processes are interlinked through talk, discourse, and the flow of information. At the social level, it is important for the receiver of a message to accept and respect the bearer of the message. Internalization theory must therefore take into account the social processes within the social context of the people when there is a wish to reach a wider audience and ensure that new groups from outside the social circle are listened to and given authority and credibility. The most effective strategy for change appears to be when the recipient obtains information from a trusted person with authority. Community-based information campaigns seem to be profitable as the meaning of the information can be discussed with others, and the pains that are felt can be shared and dealt with collectively. This is in line with lessons learned from abandonment campaigns in Africa. 

Findings from this study make it difficult to argue that attitudinal change will happen automatically merely as a consequence of staying in an FGC-negative environment, where the procedure is illegal and has low status among the majority population. Targeted information may be necessary. We know there are instances where the majority of the population is against FGC, but where a minority, like the Bohras in Pakistan, practiced it for years. The Mandinga community in the Gambia practices FGC, while the Wolof does not, in spite of coexisting in the area for centuries. For information to have an impact, the sender needs to be seen as credible and trustworthy by the receiver, and the information must be regarded as relevant. It may be that this process of acknowledging “outsiders” as a relevant public, whose information can be trusted, is taking place today in Norway and Sweden in the Somali communities there, as the study of Gele et al. [18] indicates and as the study of Johnsdotter et al. [17] has shown. If so, Somali women in both countries could be increasingly susceptible to internalize knowledge and values that will change their mind, as the authors have shown. They would then also be susceptible to the feeling of shame and the epistemological and psychological pains that we have described here, as an effect of the internalization process, when information “hit bottom.” 

### 8.1. Limitations

We do not know whether this experience of pain always accompanies a shift in attitude to FGC. It may be that the pain is present for early adopters and not for very late adopters. The data from this study indicate that a psychological and epistemological pain was part of the process of change of attitude to FGC that motivated the activists that we studied to work against the practice. It was also part of the attitudinal change process that the Gambian group experienced. But we do not know if it would be experienced by all categories of adopters of a new paradigm on FGC.

Not only verbal information works in an abandonment campaign, but songs, slides, and films addressing a wide variety of topics related to the procedure are efficient tools that can reach the innermost level of internalization, where the motivation to change behavior will be the strongest. FGC-abandonment campaigns provide interesting cases for researchers seeking to understand how people react to and deal with conflicts of values, knowledge, and traditions in the modern world. As we have seen, knowledge is not only passively received, but also positively embraced as a broadening of the mind and seen as stimulating. It can sometimes be rejected and ignored; indeed, even when it is embraced and accepted, it can be experienced as psychologically very painful. 
